# Genetic Mimicry Analysis Reveals the Specific Lipases Targeted by the ANGPTL3-ANGPTL8 Complex and ANGPTL4

**DOI:** 10.1016/j.jlr.2022.100313

**Published:** 2022-11-11

**Authors:** Fredrik Landfors, Elin Chorell, Sander Kersten

**Affiliations:** 1 Department of Public Health and Clinical Medicine, Section of Medicine, Umeå University, Umeå, Sweden; 2 Nutrition, Metabolism and Genomics group, Division of Human Nutrition and Health, Wageningen University, Wageningen, the Netherlands

**Keywords:** angiopoietin-like proteins, dyslipidemias, cardiovascular disease, lipase/endothelial, lipase/hepatic, lipidomics, lipids, lipolysis and fatty acid metabolism, lipoprotein/metabolism, triglycerides, ANGPTL, angiopoietin-like protein, ASCVD, atherosclerotic cardiovascular disease, EL, endothelial lipase, eQTL, expression quantitative trait loci, GWAS, genome-wide association study, IV, instrumental variable, LD, linkage disequilibrium, LDLR, LDL receptor, LIPC, lipase C hepatic type, LIPG, lipase G endothelial type, PCSK9, proprotein convertase subtilisin/kexin type 9, PTV, protein-truncating variant, *R*^2^, coefficient of determination, TG, triglyceride

## Abstract

Angiopoietin-like proteins, ANGPTL3, ANGPTL4, and ANGPTL8, are involved in regulating plasma lipids. In vitro and animal-based studies point to LPL and endothelial lipase (EL, *LIPG*) as key targets of ANGPTLs. To examine the ANGPTL mechanisms for plasma lipid modulation in humans, we pursued a genetic mimicry analysis of enhancing or suppressing variants in the *LPL*, *LIPG*, lipase C hepatic type (*LIPC*), *ANGPTL3*, *ANGPTL4*, and *ANGPTL8* genes using data on 248 metabolic parameters derived from over 110,000 nonfasted individuals in the UK Biobank and validated in over 13,000 overnight fasted individuals from 11 other European populations. ANGPTL4 suppression was highly concordant with LPL enhancement but not HL or EL, suggesting ANGPTL4 impacts plasma metabolic parameters exclusively via LPL. The LPL-independent effects of ANGPTL3 suppression on plasma metabolic parameters showed a striking inverse resemblance with EL suppression, suggesting ANGPTL3 not only targets LPL but also targets EL. Investigation of the impact of the ANGPTL3-ANGPTL8 complex on plasma metabolite traits via the *ANGPTL8* R59W substitution as an instrumental variable showed a much higher concordance between R59W and EL activity than between R59W and LPL activity, suggesting the R59W substitution more strongly affects EL inhibition than LPL inhibition. Meanwhile, when using a rare and deleterious protein-truncating *ANGPTL8* variant as an instrumental variable, the ANGPTL3-ANGPTL8 complex was very LPL specific. In conclusion, our analysis provides strong human genetic evidence that the ANGPTL3-ANGPTL8 complex regulates plasma metabolic parameters, which is achieved by impacting LPL and EL. By contrast, ANGPTL4 influences plasma metabolic parameters exclusively via LPL.

Atherosclerotic cardiovascular disease (ASCVD) is the leading cause of morbidity and premature death worldwide. Convincing evidence from human genetics supports a causal association between triglycerides (TGs), TG-rich lipoproteins and their remnants, and ASCVD (1). Accordingly, therapies aimed at lowering plasma TGs can be an important strategy to reduce the risk of ASCVD in certain individuals.

TG is transported in the bloodstream by TG-rich lipoproteins in the form of chylomicrons and very low density lipoproteins. The plasma concentration of TG-rich lipoproteins is determined by their rate of production in the intestine and liver, by the TG clearance rate via intravascular lipolysis in muscle and adipose tissue, and by hepatic clearance of the remnant particles (1). The intravascular lipolysis of circulating TG to fatty acids is catalyzed by the enzyme LPL and is rate limiting for TG uptake into tissues (2). To adjust TG uptake to changes in TG availability and local fatty acid demand, the activity of LPL in different tissues is carefully regulated. A group of proteins that plays an important role in the regulation of LPL activity in various tissues are the angiopoietin-like proteins (ANGPTLs), consisting of ANGPTL3, ANGPTL4, and ANGPTL8 (3, 4).

ANGPTL4 was identified as a transcriptional target of PPARs in adipose tissue and liver (5, 6). Studies in genetically modified mice have shown that ANGPTL4 reduces LPL activity and plasma TG clearance in adipose tissue during fasting, thereby directing circulating TG to other tissues (7, 8, 9, 10, 11, 12, 13, 14). In addition, ANGPTL4 regulates LPL activity in brown adipose tissue during cold (15), in skeletal muscle during exercise (16), and in the heart and macrophages after lipid loading (17, 18, 19, 20). ANGPTL4 inhibits LPL by promoting the unfolding, proteolytic cleavage, and subsequent degradation of LPL, causing a decrease in the amount of active LPL participating in TG hydrolysis (21, 22, 23). Besides inhibiting LPL, there is evidence that ANGPTL4 also inhibits HL (12, 24). Genetic studies strongly support the role of ANGPTL4 in the regulation of plasma lipid levels in humans, showing that carriers of the E40K loss-of-function variant in ANGPTL4 have lower plasma TG and higher plasma HDL-C levels than noncarriers (25, 26, 27, 28). Consistent with a causal role of TG in ASCVD, E40K carrier status is associated with a marked decrease in the risk of coronary artery disease (25, 27, 29, 30). Based on these findings, targeting ANGPTL4 has become an attractive pharmacological strategy to lower plasma levels of TG-rich lipoproteins and reduce the risk of ASCVD.

ANGPTL3 was identified as the gene mutated in a hyperlipidemic rodent model (31). In contrast to ANGPTL4, which is expressed in numerous tissues, ANGPTL3 is exclusively produced in hepatocytes (32). Studies in ANGPTL3-deficient and ANGPTL3-overexpressing mice have shown that ANGPTL3 impairs clearance of TG-rich lipoproteins and raises plasma levels of TG by inhibiting the activity of LPL (31, 33). As observed for ANGPTL4, genetic studies support a regulatory role of ANGPTL3 in controlling plasma lipid levels in humans. Specifically, carriers of loss-of-function variants in ANGPTL3 have markedly lower levels of TG and LDL-C, which is associated with a reduction in the odds of ASCVD of about 40% (34, 35).

Although initially it was thought that ANGPTL3 is a direct and independent inhibitor of LPL, the relatively weak inhibition of LPL by ANGPTL3 hinted at a more complex mechanism of action (36). The missing piece of the puzzle turned out to be ANGPTL8. ANGPTL8 is a truncated member of the ANGPTL family that is produced in hepatocytes and adipocytes and is highly inducible by insulin (37, 38, 39). Studies in mice showed that the ability of ANGPTL3 to raise plasma TG depends on ANGPTL8 (37). In vitro studies subsequently found that ANGPTL3 is released from liver cells partly as a complex with ANGPTL8 (40, 41). The physical association between ANGPTL3 and ANGPTL8 creates a potent inhibitor of LPL that impairs plasma TG clearance, with a preference for the heart, skeletal muscle, and brown adipose tissue (42). Whereas nearly all ANGPTL8 secreted by hepatocytes is complexed to ANGPTL3, most of the ANGPTL3 released and present in blood plasma is unbound (43). Besides inhibiting LPL, ANGPTL3 also inhibits endothelial lipase (EL). It was recently shown that in the absence of LDL receptor (LDLR), the lowering of plasma LDL-C by ANGPTL3 deficiency is dependent on EL (44, 45). Currently, conflicting data exist about whether unbound ANGPTL3 or the ANGPTL3-ANGPTL8 complex is a more potent inhibitor of EL (46, 47).

Genetic mimicry analysis is a tool to unveil enzyme regulatory pathways via the use of genetic instrumental variables (IVs) derived from large-scale genome-wide association study (GWAS). This method has been used to evaluate pathway specificities of drug therapies in the LDLR pathway. As proof of principle, the systemic effects of functional genetic variation in HMG-CoA reductase (*rs12916-T*) showed almost perfect mimicry to statin therapy (48). In addition, proprotein convertase subtilisin/kexin type 9 (PCSK9) inhibition proxied through *PCSK9 rs11591147-T* was largely concordant with pravastatin therapy (49).

Recently, a genetic mimicry analysis was also conducted on ANGPTL3 and ANGPTL4 suppression. This study confirmed the role of LPL in the metabolic effects of ANGPTL3 and ANGPTL4 in humans (50). Although in vitro experiments and studies in genetically modified mice strongly suggest that ANGPTL3 raises plasma TG via complex formation with ANGPTL8; this notion has yet to be confirmed by genetic epidemiology. In addition, genetic epidemiological approaches have not yet been applied to assess the regulation of EL by ANGPTL3 and ANGPTL8.

Here, we pursued a genetic mimicry analysis using data derived from over 120,000 individuals in the UK Biobank and 11 other European populations to examine the mechanism of plasma lipid regulation by ANGPTLs in humans. Our results strongly suggest that the ANGPTL3-ANGPTL8 complex inhibits both LPL and EL. Furthermore, our data suggest that ANGPTL4 impacts plasma lipids exclusively via LPL, whereas ANGPTL3 acts through both LPL and EL. By specifically activating the LPL pathway, ANGPTL4 suppression may confer the same cardiometabolic benefits as gain-of-function variants in the *LPL* gene.

## Materials and Methods

### Ethical review

The analyses were performed using anonymized GWAS summary statistics from previous studies (26, 51, 52, 53, 54, 55, 56, 57, 58). A separate institutional ethical review was not required for this study. The studies abode to the Declaration of Helsinki Ethical Principles for Medical Research Involving Human Subjects and were approved by appropriate institutional ethical review boards (26, 51, 52, 53, 54, 55, 56, 57, 58).

### Study design and data sources

A descriptive summary of the datasets with study designs, genotyping method, NMR platforms, and nationalities is given in Table 1. The derivation cohort included a subsample of the UK Biobank of up to 115,078 individuals whose plasma had been metabolically profiled (*N* parameters = 248) using a quantitative NMR metabolomics platform (51). The UK Biobank is a population-based longitudinal study that recruited over 500,000 individuals between the year 2006 and year 2010 (59). For the validation analysis, we retrieved data from a meta-analysis of 11 GWASs that included up to 24,925 individuals and 122 NMR parameters (52). The validation set substudies used population-based cohort, case-control, birth cohort, family based, and twin study designs and were performed in Dutch, Estonian, Finnish, and German populations. The UK Biobank derivation cohort participants were not instructed to fast overnight. In contrast, the overwhelming majority of the validation set participants were instructed to fast overnight. In both cases, the GWAS genotype effect estimation procedure involved correction for either fasting status, time since last meal, or both. This means that the UK Biobank participants should be considered as nonfasted, whereas the validation set participants are to be considered overnight fasted. The derivation and validation set studies used two different versions of a commercial NMR metabolomics platform (Nightingale Health Ltd, Helsinki, Finland). The platform measures over a hundred plasma metabolite parameters, including free fatty acids, amino acids, lipoprotein size, lipoprotein subclass lipid concentrations, and lipoprotein subclass lipid composition (60). The quality control and preprocessing procedures for the derivation cohort NMR measurements have been described previously (61). The method has been widely applied in epidemiological studies (62). Detailed definitions of the 248 plasma parameters measured by NMR in the derivation population (supplemental Table S1) and 122 parameters in the validation populations (supplemental Table S2) are given in the supplemental appendix. As shown in Table 1, common and low-frequency variant data (allele frequency >1%) were retrieved from microarray-based GWASs (51, 52). As very rare (allele frequency <0.1%) variants are not commonly available from the microarray-based methods, data on the very rare ANGPTL8 protein-truncating variant (PTV) (allele frequency = 0.04%) were retrieved from a whole-exome sequencing association analysis of the UK Biobank cohort (55).

**Table 1:** Description of GWAS datasets

	Derivation cohort	Validation set
Data source	UK Biobank (UKBB)	Meta-analysis of 11 European studies
Study design	Population-based cohort	Population-based cohorts, case-control studies (only controls used), birth cohort, family based studies (adjusted for family structure), and twin studies (adjusted for relatedness)
Population	British	Dutch, Estonian, Finnish, and German
Sample size per metabolic parameter	110,058–115,078	13,171–24,925
Fasting status	Nonfasted	Fasted
Genotyping platform(s)	UKBB axiom array/IDT xGen Exome Research Panel v1.0 w. Illumina NovaSeq 6000 platform	Affymetrix 6.0, Affymetrix 250K, Affymetrix 6.0 907K, Illumina 318K, Illumina 370K, Illumina 610K, Illumina 660K, Illumina 670K, Illumina CoreExome, Illumina Human660W, Illumina HumanCNV370, Illumina HumanOmniExpress, Illumina Omni 1M, Illumina OmniExpress, and Perlegen-Affymetrix 500K
Number of SNPs	12,321,875/2,043,019	11,274,684–12,092,490
Metabolic parameter analysis platform(s)	500 MHz Bruker AVANCE III HD	500 MHz Bruker AVANCE III HD, 600 MHz Bruker AVANCE II
Number of metabolic parameters	248	122

### Genetic instrument justification

The genetic IVs used in the genetic mimicry analyses need to follow the core assumptions of IV analysis (see the *Technical note* in the supplemental appendix for details). Each genetic variant must 1) be associated with altered biologic activity of the target protein, 2) not be associated with any confounders of the protein-metabolite associations, and 3) only be associated with the metabolites through its effect on the target protein. To satisfy these assumptions, LPL, EL, HL, ANGPTL3, ANGPTL4, and ANGPTL3-ANGPTL8 complex enhancement or suppression were proxied by either 1) missense mutations that cause gain or loss of function in the target protein, 2) nonsense/stop-gain mutations that introduce a premature stop codon, or by 3) variants in or near cis-acting loci that were associated with increased or decreased gene transcription in biologically relevant tissues. The latter can be referred to as local- or cis-expression quantitative trait loci (eQTL). We chose the same cis-eQTL variants for LPL and ANGPTL3 as a previous genetic mimicry study that compared ANGPTL3 and ANGPTL4 inhibition of LPL (50). To validate them, we ran colocalization analyses of GWAS summary statistics of serum TGs (52, 54), with tissue gene expression data from GTEx Analysis Release V7 (dbGaP accession: phs000424.v7.p2 (53)). The colocalization analyses provides a probability measure that two genetic traits share the same single causal variant, for example, the probability that an *LPL* variant affects both gene expression and serum TGs (63). We used a probability threshold of ≥75% posterior probability (Prob(H_4_)) to conclude that a single variant affected both traits. Also, we controlled that the IVs were not confounded by linkage disequilibrium (LD) via the use of the National Institutes of Health National Cancer Institute Division of Cancer Epidemiology and Genetics LDproxy tool (64). We plotted coefficient of determination (*R*^2^) values within a 1 megabase pair window using a European reference population and determined if there was significant LD (*R*^2^ ≥ 0.1) with variants in neighboring genes.

### Statistical analysis

The degree of mimicry between LPL, EL, HL, ANGPTL3, ANGPTL4, and ANGPTL3-ANGPTL8 complex effect-altering variants were compared by calculating the conditional explained variance using the *R*^2^ by linear regression. The effect estimates of lipid parameters from the first genetic IV were regressed upon the effect estimates from the second genetic IV (see the *Technical note* in the supplemental appendix for details). The models assumed additive interaction among alleles for the directionality of effects. This is different from additive interaction for absolute effects. For example, genetic disorders that follow an autosomal recessive pattern of inheritance because of nonadditive interaction among alleles for absolute effects (e.g., primary LPL deficiency [biallelic *LPL null*]) would still satisfy the assumption if the effect size directionality (e.g., using two parameters: higher TGs and proportionally lower HDL cholesterol per 1-SD elevated TGs) was concordant between monoallelic and biallelic *LPL null* carriers. Analyses were conducted separately for the derivation cohort and the validation set. To ease comparisons between the IV targets (e.g., LPL activity enhancement and ANGPTL4 suppression), each effect estimate was scaled so that it represented the 1-SD effect on each parameter per 1-SD effect on total TGs or total cholesterol. The scaling does not affect the statistical parameter of interest (*R*^2^). 1-SD change of total TGs was approximately equal to 0.57 mmol/l, and 1-SD change of total cholesterol was approximately equal to 0.94 mmol/l. The regression slope CIs are presented with Bonferroni multiple comparison correction. We tested nine independent hypotheses. Therefore, each 95% CI was adjusted to the level of $1-\frac{0.05}{9}$. The statistical analyses were performed using R software (The R Foundation, version 4.1.1).

## Results

### Validation of genetic instruments

A descriptive summary of the genetic instruments is given in Table 2. LPL activity enhancement was proxied using *LPL rs115849089-A*. The *rs115849089-A* variant is located ∼153,000 base pairs downstream from the *LPL* transcription start site and is associated with increased expression of *LPL* in whole blood (normalized effect size ≈ 0.51, *P* ≈ 1.21 × 10^−7^). Colocalization analysis using variants within a 200,000 base-pair window of rs115849089 provided a 70% posterior probability of plasma TG and LPL expression sharing a single causal variant (supplemental Fig. S1). As the colocalization test was inconclusive (Prob(*H*_*4*_) <75%), we conducted a sensitivity analysis using the functional variant *LPL rs1801177*-*A* in the derivation cohort. The mutation leads to a D36N substitution that causes a ∼20% reduction in specific LPL activity (71). Our sensitivity analysis showed that the systemic effects of *LPL rs115849089-A* and *LPL rs1801177*-A were strongly inversely correlated with each other (supplemental Fig. S2: *R*^2^ ≈ 0.96, slope ≈ −0.94 [95% CI: −0.97, −0.90], intercept ≈ −0.05 [95% CI: −0.08, −0.03]). Also, the main results remained intact when instrumenting LPL activity through *rs1801177*-A (supplemental Fig. S3). These results confirm that *LPL rs115849089-A* is a valid target for genetically instrumenting LPL enhancement. It was not possible to conduct sensitivity analyses of concordance between rs1801177-A and rs115849089-A in the validation set. The validation set allele frequency was ∼0.4%, which meant there were only up to 51–121 *rs1801177-A* carriers per metabolic parameter. In contrast, the allele frequency was ∼1.7% in the larger UK Biobank cohort, which amounted to up to 1902–1989 *rs1801177-A* carriers per metabolic parameter.

**Table 2:** Instrument justification for genetic variants

Protein target	Genetic variant used for instrumentation (chr:pos)	Allele frequency (derivation/validation set)	Mutation consequence	Effect on clinical phenotypes^a^ (β/OR^b^; *P* value (H_0_))
LPL	rs115849089-A (8:19,912,370)	11.4–11.5%/10.0–11.2%	*Increases the expression of LPL*: Located ∼153,000 base pairs downstream of the *LPL* gene. eQTL for LPL RNA expression in whole blood (supplemental Fig. S1)	TG: −0.18; ≤2 × 10^−308^ HDL: 0.16; *≤*2 × 10^−308^ LDL: −0.01; 6 × 10^−5^ CAD: 0.95; 4 × 10^−9^
ANGPTL3	rs11207977-T (1:62,977,307)	35.0–35.1%/26.9%–29.0%	*Decreases the expression of ANGPTL3*: *DOCK7* intronic variant located ∼86,000 base pairs upstream of *ANGPTL3*. eQTL for ANGPTL3 RNA expression in hepatic tissue (supplemental Fig. S4)	TG: −0.08; 2 × 10^−304^ HDL: −0.02; 2 × 10^−13^ LDL: −0.04; 7 × 10^−69^ CAD: 0.99; 0.69
ANGPTL4	rs116843064-A (19:8,429,323)	2.0%/2.9–3.1%	*Alters the function of ANGPTL4*: Missense variant that causes the E40K substitution. This destabilizes the protein after secretion and prevents ANGPTL4 inhibition of LPL (65)	TG: −0.22; 1 × 10^−16^ HDL: 0.21; 3 × 10^−195^ LDL: 0; 0.9 CAD: 0.87; 4 × 10^−10^
ANGPTL8	rs2278426-T (19:11,350,488)	3.5%/5.7–6.5%	*Alters the function of ANGPTL3-ANGPTL8 complex:* Missense variant that causes a R59W replacement. In vitro studies suggest that ANGPTL3-ANGPTL8 R59W have a decreased ability to bind and inhibit LPL, when compared with wild-type ANGPTL3-ANGPTL8 (47)	TG: −0.04; 1 × 10^−14^ HDL: −0.09; 3 × 10^−69^ LDL: −0.04; 4 × 10^−15^ CAD: 1.01; 0.25
ANGPTL8	rs145464906-T (19:11,350,874)	0.04%	PTV*:* Nonsense Q121X mutation that results in a premature stop codon. Truncated ANGPTL8 does not form complex with ANGPTL3 (66)	TG: −0.54; 2 × 10^−33^ HDL: 0.44; 3 × 10^−22^ LDL: −0.15; 2 × 10^−3^ CAD: 0.73; 0.21
EL (*LIPG*)	rs77960347-G (18:47,109,955)	1.3%/0.5–0.7%	*Alters the function of EL:* Missense mutation causing the N396S substitution. Leads to a 40% decrease of EL activity in vitro (67)	TG: 0.03; 5 × 10^−4^ HDL: 0.29; 4 × 10^−269^ LDL: 0.07; 8 × 10^−15^ CAD: 0.90; 9 × 10^−5^
HL (*LIPC*)	rs1800588-T (15:58,723,675)	21.4%–21.6%/23.5–25.0%	*Decreases the expression of HL:* Promoter variant associated with ∼30% lower *LIPC* promoter activity in cell lines and ∼15% lower HL activity in human postheparin plasma (68, 69, 70)	TG: 0.05; 2 × 10^−84^ HDL: 0.12; ≤2 × 10^−308^ LDL: 0.02; 5 × 10^−17^ CAD: 1.03; 2 × 10^−5^

CAD, coronary artery disease; Chr:pos, genomic location as chromosome:position using Genome Reference Consortium Human Build 37 (GRCh37/hg19); H0, null hypothesis; HDL, cholesterol in HDL; LDL, cholesterol in LDL; TG, total TGs.

a Lipid (TG, LDL-C, and HDL-C) and CAD data summary statistics were obtained from Refs. (56) and (57), respectively. The ANGPTL8 PTV summary statistics were obtained from Refs. (26, 58).

b β-coefficients/slope indicating the 1-SD effect of the effect allele are presented for the continuous clinical lipid outcome measures. Odds ratios (ORs) were used for CAD.

ANGPTL3 suppression was surrogated using *ANGPTL3 rs11207977-T*. This variant is an eQTL that is associated with increased *ANGPTL3* transcription in the liver (normalized effect size ≈ −0.32, *P* ≈ 1.16 × 10^−7^). Colocalization analysis using a 200,000 base-pair window suggested a 94.1% posterior probability of *ANGPTL3* liver expression and circulating TG sharing a single causal variant (supplemental Fig. S4). ANGPTL4 suppression was proxied using *ANGPTL4 rs116843064-A*. This missense mutation causes the E40K substitution, which destabilizes ANGPTL4 and thus decreases effective LPL inhibition (65).

ANGPTL3-ANGPTL8 complex action was surrogated by two different variants: 1) the *ANGPTL8 rs2278426-T* common variant, which causes the R59W substitution and 2) the *ANGPTL8 rs145464906-T*, a very rare predicted PTV, which introduces a Q121X stop-gained/nonsense mutation. In vitro LPL activity and LPL binding assays indicate that the R59W substitution decreases the ability of ANGPTL3-ANGPTL8 complexes to bind and inhibit LPL (47). *ANGPTL8* rs2278426 is located ∼0.1 megabase pairs downstream of the *LDLR* gene. Because of the proximity of *ANGPTL8* and *LDLR*, confounding by LD with *LDLR* variants is possible. However, none of the variants in significant LD with *ANGPTL8* rs2278426 were significant eQTLs for liver LDLR expression (supplemental Table S3). Also, there was no significant LD of *ANGPTL8* rs2278426 with exonic *LDLR* variants (supplemental Fig. S5). The *Q121X* nonsense mutation is a very rare variant with an allele frequency of 0.04% in the UK Biobank derivation cohort. Nonsense mutations can result in decreased or altered protein function via two mechanisms: 1) translation of a truncated protein resulting in either gain or loss of function and 2) decreased transcription via nonsense-mediated mRNA decay (72). Supporting the notion that this variant could be used as a surrogate for ANGPTL3-ANGPTL8 complex action, an in vitro study found that truncated ANGPTL8 does not form complex with ANGPTL3 and therefore cannot inhibit LPL (66).

EL suppression was proxied by the missense mutation lipase G endothelial type (LIPG) *rs77960347-G*, which causes the N396S substitution. In vitro studies found this mutation to be associated with a 40% decrease in EL activity (67). The decreased lipase activity was confirmed in a rodent model carrying the N396S substitution, which was concordant with the increased HDL-C levels found in humans (67). HL activity inhibition was instrumented through *LIPC rs1800588-T.* This variant is located in the *LIPC* promotor region at position 514. It is associated with a ∼30% reduction of *LIPC* promoter activity in vitro and ∼15% lower HL activity as determined by postheparin plasma assays in humans (68, 69, 70).

### LPL mimicry patterns of ANGPTL3, ANGPTL4, and ANGPTL3-ANGPTL8 complexes

**Fig. 1:**
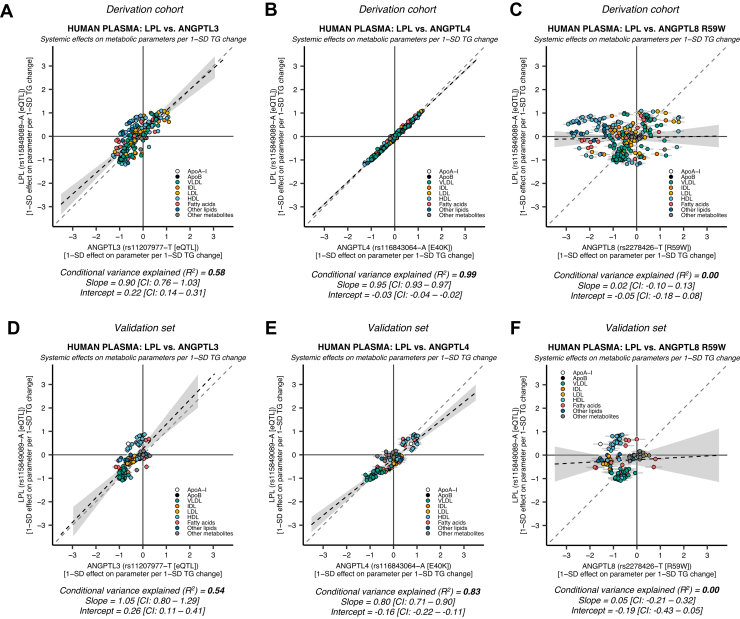
**ANGPTL3-, ANGPTL4-, and*****ANGPTL8*****R59W-mediatedgenetic LPL disinhibition shows distinct patterns of LPL mimicry.** The similarity of genetic LPL enhancement and ANGPTL3, ANGPTL4, or ANGPTL3-ANGPTL8 complex suppression was compared using data derived from up to 115,078 individuals on 248 metabolic parameters in the UK Biobank (derivation cohort) and validated in up to 24,925 individuals on 122 metabolic parameters in an independent European validation set. A: LPL enhancement versus ANGPTL3 suppression in the derivation cohort. B: LPL enhancement versus ANGPTL4 suppression in the derivation cohort. C: LPL enhancement versus an ANGPTL3-ANGPTL8 complex function altering variant (ANGPTL8 R59W) in the derivation cohort. D: LPL versus ANGPTL3 in the validation set. E: LPL versus ANGPTL4 in the validation set. F: LPL versus ANGPTL8 R59W in the validation set. Each scatter dot represents the effect of the genetic variant on a lipoprotein lipid or plasma metabolite. The gray solid lines starting from each dot indicate the standard error of the estimate. The black dashed line indicates the regression estimate. The 95% CI of the regression is indicated by the gray area adjacent to the line. The gray dashed line indicates a reference regression line with a coefficient = 1. To ease comparisons, each effect estimate for the variant effect on each metabolic parameter was scaled so that they represent the 1-SD effect per 1-SD effect on total plasma TGs. The mimicry of effects was estimated using linear regression. The conditional variance explained (*R*^2^) indicates the degree of LPL enhancement that is explained by genetic variation in *ANGPTL3*, *ANGPTL4*, or *ANGPTL8* R59W.

The effects of genetic variants in *LPL*, *ANGPTL3*, *ANGPTL4*, and *ANGPTL8* on 248 lipoprotein lipid, free fatty acid, amino acid, and other plasma metabolite parameters in the derivation cohort, and 122 parameters in the validation set, were compared using linear regression. A detailed list of the genetic IV effects on all plasma parameters is given in supplemental Tables S4–S6. Their respective mimicry patterns are presented in Fig. 1.

The different pathway specificities of genetically proxied ANGPTL3 and ANGPTL4 suppression have been described previously (50). In concordance with this previous study, we found that ANGPTL3 suppression showed a moderate degree of LPL mimicking (Fig. 1A: *R*^2^ ≈ 0.58, slope ≈ 0.90 [95% CI: 0.76, 1.03], intercept ≈ 0.22 [95% CI: 0.14, 0.31]). The results were very similar in the validation set (Fig. 1D: *R*^2^ ≈ 0.54, slope ≈ 1.05 [95% CI: 0.80, 1.29], intercept ≈ 0.26 [95% CI: 0.11, 0.41]). Also in agreement, ANGPTL4 suppression proxied through ANGPTL4 E40K was strikingly similar to LPL enhancement in the derivation (Fig. 1B: *R*^2^ ≈ 0.99, slope ≈ 0.95 [95% CI: 0.93, 0.97], intercept ≈ −0.03 [95% CI: −0.04, −0.02]), and validation set (Fig. 1E: *R*^2^ ≈ 0.83, slope ≈ 0.80 [95% CI: 0.71, 0.90], intercept ≈ −0.16 [95% CI: −0.22, −0.11]). In a sensitivity analysis, we wanted to confirm that the systemic effects of *ANGPTL4 rs116843064-A* were a common feature of genetic variation in *ANGPTL4*, and not just specific to the E40K interaction with LPL. To do this, we conducted mimicry analyses of LPL enhancement using all the available variants within a 0.2 Mb window of *ANGPTL4* that were associated with any of the 248 parameters at a genome-wide significance level in the derivation cohort (*N* = 228 SNPs). We found a high concordance between LPL enhancement and the metabolite-associated *ANGPTL4* variants. This was not because of LD with *ANGPTL4 rs116843064-A* (supplemental Fig. S6: median *R*^2^ ≈ 0.93, interquartile range ≈ 0.86–0.94, and range ≈ 0.79–0.99). In line with the previous study (50), these results strongly suggest that ANGPTL4 suppression is more specific for the LPL activity pathway compared with ANGPTL3 suppression.

The consequences of ANGPTL8 R59W on ANGPTL3-ANGPTL8 complex inhibition of LPL and EL were recently described in both in vitro and in vivo experimental models (47). We investigated if the decreased ability of ANGPTL8 R59W to bind and inhibit LPL could elucidate the function of the ANGPTL3-ANGPTL8 complex in a human genetic epidemiologic setting. We found that the systemic effects of the ANGPTL8 R59W mutation did not mimic LPL activity enhancement or inhibition in the derivation (Fig. 1C: *R*^2^ ≈ 0.00, slope ≈ 0.02 [95% CI: −0.10, 0.13], intercept ≈ −0.05 [95% CI: −0.18, 0.08]) and validation sets (Fig. 1F: *R*^2^ ≈ 0.00, slope ≈ 0.05 [95% CI: −0.21, 0.32], intercept ≈ −0.19 [95% CI: −0.43, 0.05]). These results suggest that the univariate analysis did not capture the action of the ANGPTL3-ANGPTL8 complex on LPL, indicating that we had to try other approaches.

### Multivariable LPL mimicry analysis using ANGTPL3 and ANGPTL8 variants

**Fig. 2:**
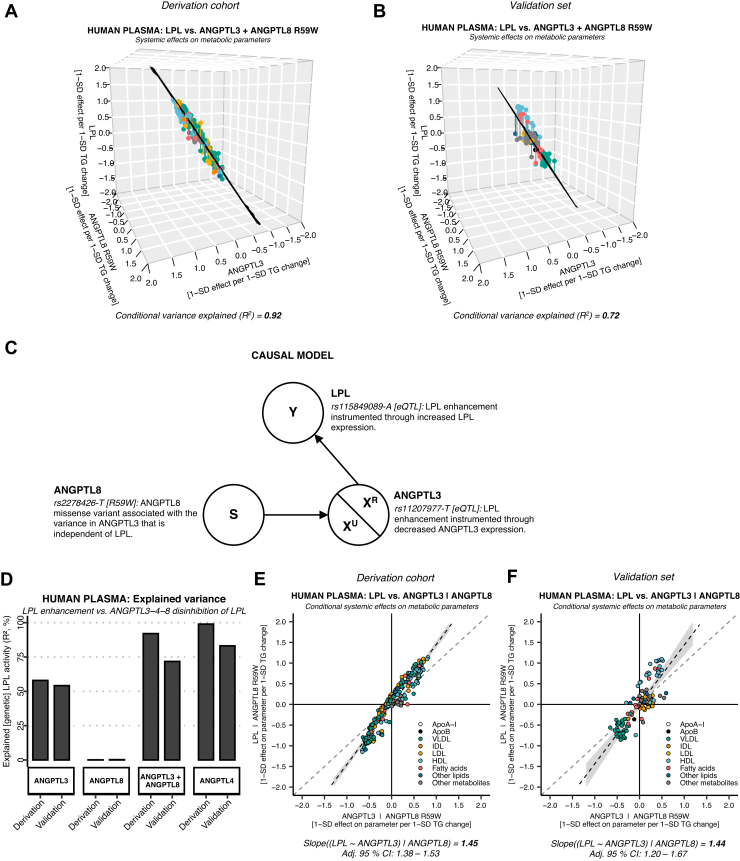
**ANGPTL3 suppression adjusted for the effects of the*****ANGPTL8*****R59W coding mutation shows a remarkably high degree of LPL mimicry.** A: Multivariable model of *ANGPTL3 rs11207977-T* and *ANGPTL8* rs2278426 *[R59W]* in the UK Biobank cohort (*N* = 110,058–115,078, NMR parameters = 248) showed a mimicry of 92% (*R*^2^). B: The pattern of increased explained variance was concordant with the European validation set (*N* = 13,171–24,925, NMR parameters = 122), with 72% conditional variance explained (*R*^2^). C: The directed acyclic graph (DAG) summarizes the causal model for the regression analysis. In ANGPTL3, there was variance that is related to LPL enhancement (X^R^) and variance unrelated to LPL enhancement (X^U^). D: Bar plot comparing the explained variance of each genetic instrument. X^R^ in ANGPTL3 (instrumented through decreased *ANGPTL3* transcription) was 55–58%, whereas the X^R^ of altered ANGPTL3-ANGPTL8 complex function was 0% (instrumented through the *ANGPTL8* R59W mutation). However, when correcting for the ANGPTL3-ANGPTL8-mediated LPL disinhibition in an ANGPTL3 + ANGPTL8 model, the proportion of conditional variance explained increased to 72% and 92%. E: The coefficient of ANGPTL3 suppression accounting for ANGPTL8 R59W increased from 0.90 to 1.45 in the derivation cohort. F: The pattern was replicated using the same method in the validation set (unconditional coefficient = 1.05, conditional coefficient = 1.44). Together, this suggested that *ANGPTL8 rs2278426-T* [R59W] acts as a statistical suppressor (73). This means that it is correlated to, and corrects for, the unrelated variance (X^U^) in the relationship between ANGPTL3 and LPL. In this case, X^U^ should be read as the systemic effects of decreased ANGPTL3 transcription via pathways unrelated to LPL (and random error).

To further investigate the role of the ANGPTL3-ANGPTL8 complex, we analyzed the relationship of LPL versus ANGPTL3 and ANGPTL8 R59W using multivariable linear regression. We found that the combined effects of ANGPTL3 suppression and ANGPTL8 R59W mimicked LPL enhancement almost as well as ANGPTL4 suppression (Fig. 2A: *R*^2^ ≈ 0.92). We saw the same pattern in the validation set, even though the *R*^2^ was slightly smaller (Fig. 2B: *R*^2^ ≈ 0.72 and Fig. 2D). This discrepancy may be explained by smaller statistical power (greater random error when estimating the effects on individual parameters) and heterogeneous sampling in the different subcohorts (i.e., greater measurement error). To investigate the conditional effects of each variant, we plotted the relationship between LPL and the ANGPTLs using added-variable plots. By adding ANGPTL8 R59W to the model, the ANGPTL3 coefficient increased from 0.90 to 1.45 in the derivation cohort (Fig. 2E) and 1.05 to 1.44 in the validation set (Fig. 2F). We consider this strong evidence for a statistical suppressor effect (73). This means that ANGPTL8 R59W increased the conditional explained variance (*R*^2^) through its association with the residual error of the ANGPTL3-LPL relationship (see technical details in Fig. 2C). Next, we aimed to identify the cause of the ANGPTL3-LPL residuals and to find out why ANGPTL8 R59W was correcting this error.

### Genetic evidence for ANGPTL3-ANGPTL8 complex inhibition of EL

**Fig. 3:**
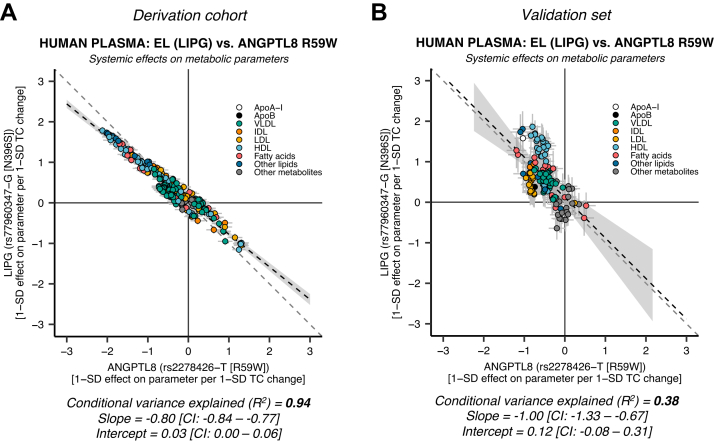
**The ANGPTL8 R59W missense mutation shifts the inhibitory action of ANGPTL3-ANGPTL8 complexes from LPL to EL (*****LIPG*****).** A: EL versus ANGPTL8 R59W in the European validation set (*N* = 110,058–115,078, NMR parameters = 248). The systemic effects of the ANGPTL8 R59W substitution strongly resemble the effects of EL suppression (*R*^2^ = 94%). B: EL versus ANGPTL8 R59W in the European validation set (*N* = 13,171–24,925, NMR parameters = 122). The coefficients, but not the conditional variance explained (*R*^2^ = 38%), were replicated in the validation set analysis. The EL validation set results should be interpreted with caution. There were only up to 66–175 LIPG rs77960347-G carriers per metabolic parameter (for allele frequency, see Table 2), which could lead to statistical imprecision. Since EL inhibition had a small effect on total TG levels (Table 2), the effect estimates were scaled so that they represent the 1-SD effect per 1-SD total cholesterol (TC) change (in contrast to Figs. 1 and 2). The different scaling does not influence the conditional explained variance (*R*^2^).

Besides inhibiting LPL, there is convincing evidence that ANGPTL3 also inhibits EL, either alone or as part of the ANGPTL3-ANGPTL8 complex (44, 45, 46, 47, 74, 75). Therefore, we hypothesized that genetically proxied EL activity could be the missing piece to our partially incomplete model. The EL-activity suppressing variant *LIPG rs77960347* was used to investigate the concordance of EL suppression with ANGPTL8 R59W. We found that the systemic effects of the R59W mutation were highly concordant with genetic EL suppression in a univariate model (Fig. 3A: *R*^2^ ≈ 0.94, slope ≈ −0.80 [95% CI: −0.84, −0.77], intercept ≈ 0.03 [95% CI: 0.00, 0.06]). The coefficients and intercepts, but not the *R*^2^, were replicated in the validation set (Fig. 3B: *R*^2^ ≈ 0.38, slope ≈ −1.00 [95% CI: −1.33, −0.67], intercept ≈ 0.12 [95% CI: −0.08, 0.31]). The nonreplicability of the *R*^2^ in the validation set may be explained by statistical imprecision because of the combination of weak phenotypic effects and a relatively low R59W coding variant prevalence (see Fig. 3 legend for technical details). These data indicate that the R59W mutation leads to higher EL activity. Interestingly, a much higher concordance was found between ANGPTL8 R59W and EL activity than between ANGPTL8 R59W and LPL activity, suggesting that ANGPTL8 R59W more strongly affects EL inhibition by ANGPTL3–-ANGPTL8 than LPL inhibition.

### ANGPTL8 PTV shows very high concordance with LPL enhancement

**Fig. 4:**
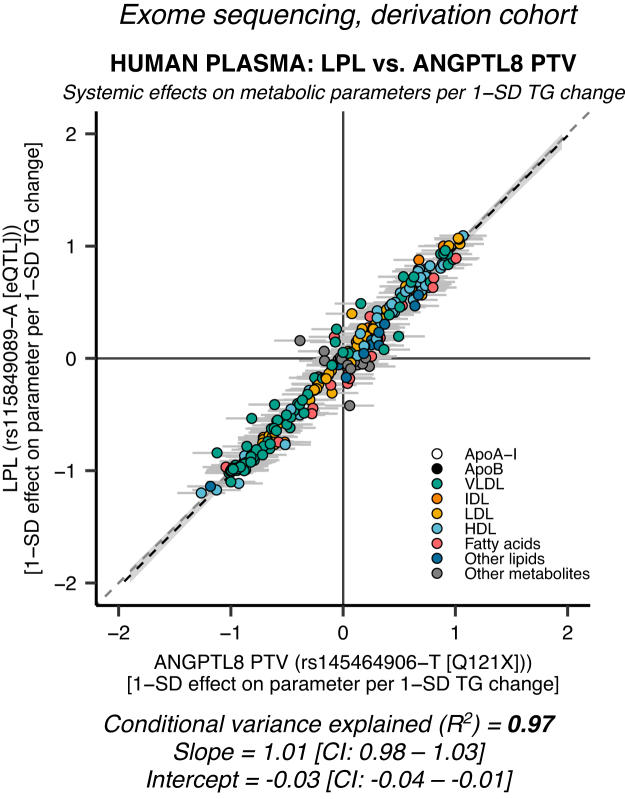
**An*****ANGPTL8*****PTV is highly LPL specific.** Data on the very rare (allele frequency = 0.04%) *ANGPTL8* Q121X coding variant was obtained from an exome sequencing analysis of the derivation cohort (*N* = 110,058–115,078). PTVs introduce a premature stop codon that disrupts transcription and causes the translation of a shortened protein. The figure shows the effects of an LPL-enhancing eQTL versus the *ANGPTL8* Q121X PTV on 248 metabolic parameters. The degree of LPL mimicry was remarkedly high (*R*^2^ = 97%) and comparable to the LPL specificity of ANGPTL4 (Fig. 1B, E). These results strongly suggest that ANGPTL3-ANGPTL8 complexes act on plasma lipids through inhibition of LPL.

We found that ANGPTL8 R59W very likely acts through EL (Fig. 3A, B). However, our finding could be explained by ANGPTL8 R59W affecting this specific pathway more than others. Thus, we performed a genetic mimicry analysis using the very rare *ANGPTL8 rs145464906-T* Q121X PTV. The PTVs have good a priori justification for utilization as genetic IVs, since they disrupt transcription and have deleterious effects on protein levels. Effect estimate data for the Q121X PTV on 248 different NMR metabolic parameters in 110,058–115,078 individuals were available from a recent whole exome sequencing effort in the UK Biobank (55). We found that ANGPTL3-ANGPTL8 suppression surrogated through the *ANGPTL8* Q121X coding variant showed a very high degree of LPL mimicry (Fig. 4: *R*^2^ ≈ 0.97, slope ≈ 1.01 [95% CI: 0.98–1.03], intercept ≈ −0.03 [95% CI: −0.04, −0.01]). In contrast to the *ANGPTL8* R59W coding variant, the LPL concordance of *ANGPTL8* Q121X was on par with *ANGPTL4* *E40K* (Fig. 1B, E). These results imply that ANGPTL3-ANGPTL8 complexes act on plasma lipids through inhibition of LPL.

### The LPL-ANGPTL3 residual variance is explained by ANGPTL3 action through EL

**Fig. 5:**
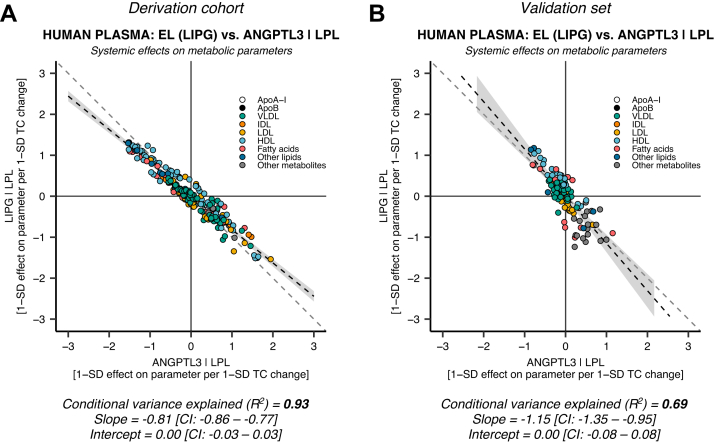
**Genetic evidence for ANGPTL3 inhibition of both LPL and EL.** The similarity of EL (*LIPG*) suppression was compared with the residuals from the LPL versus ANGPTL3 model (Fig. 1A, D) in the derivation (*N* = 110,058–115,078, NMR parameters = 248) and validation sets (*N* = 13,171–24,925, NMR parameters = 122). A: EL (*LIPG*) suppression explains the variance in ANGPTL3 that is unrelated to LPL enhancement in the derivation set (X^U^, Fig. 2), with an explained conditional variance (*R*^2^) of 93%. B: The EL versus ANGPTL3 conditional on LPL results were replicated in the European validation set; however, with a smaller *R*^2^ of 69%. These data confirm that ANGPTL3 affects plasma lipids through both LPL and EL.

The moderate concordance of ANGPTL3 suppression with LPL enhancement in this and previous studies (50) suggests that ANGPTL3 is involved in other pathways than LPL. Accordingly, we hypothesized that the lesser LPL mimicry of ANGPTL3 compared with ANGPTL4 could be explained by ANGPTL3 residual action via the EL activity pathway. We calculated the LPL-independent EL mimicry of ANGPTL3 by regressing the systemic effects of EL suppression on the residuals from the ANGPTL3-LPL model. They were highly concordant with EL suppression in the derivation set (Fig. 5A: *R*^2^ ≈ 0.93, slope ≈ −0.81 [95% CI: −0.86, −0.77], intercept ≈ 0.00 [95% CI: −0.03, 0.03]). The coefficients were replicated in the validation set (Fig. 5B: *R*^2^ ≈ 0.69, slope ≈ −1.15 [95% CI: −1.35, −0.95], intercept ≈ 0.00 [95% CI: −0.08, 0.08]). This genetic evidence further suggests that the systemic effects of ANGPTL3 suppression are mediated by both LPL and EL.

### No evidence for ANGPTL4 influence on plasma lipids through HL or EL

**Fig. 6:**
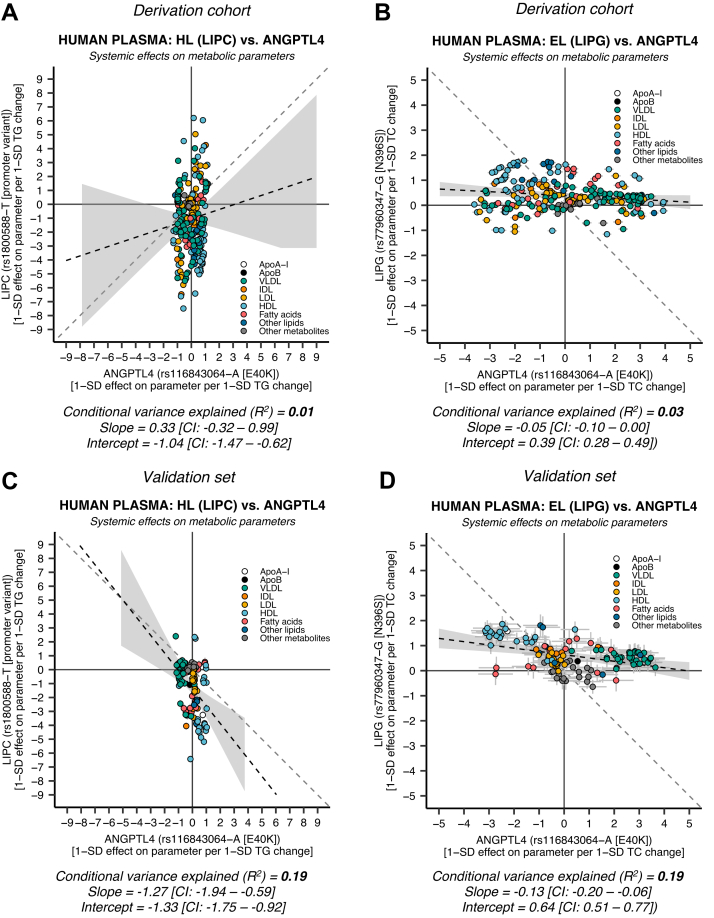
**HL (*****LIPC*****) and EL (*****LIPG*****) inhibition show minimal concordance with ANGPTL4 suppression.** HL activity was instrumented through *LIPC* rs1800588-T, a promoter variant associated with decreased *LIPC* promoter activity and lower postheparin HL activity. EL inhibition was instrumented using *LIPG rs77960347-G*, a missense mutation (N396S) leading to decreased EL activity. A: HL versus ANGPTL4 suppression in the derivation cohort using data on 248 NMR metabolic parameters derived from 110,058 to 115,078 UK Biobank individuals. B: EL versus ANGPTL4 suppression in the derivation cohort. C: HL versus ANGPTL4 suppression using data on 122 NMR metabolic parameters derived from 13,171 to 24,925 in the non-UK European validation set. D: EL versus ANGPTL4 suppression in the validation set. Because EL had a small effect on TG levels (Table 2), the EL effect estimates were scaled so that they represent 1-SD effect per 1-SD total cholesterol (TC) change (in contrast to HL and LPL). The different scaling has no effect on the conditional explained variance (*R*^2^).

Hepatic ANGPTL4 expression has been implicated in the regulation of HL (12, 24). Also, ANGPTL4 was found to be an effective EL inhibitor in vitro (46). We pursued mimicry analysis of HL (*LIPC*) and EL (*LIPG*) activity altering versus genetic variation in *ANGPTL4* to examine if there was any evidence for ANGPTL4 action on plasma lipids through HL or EL. We found a minimal correlation between the systemic effects of HL inhibition and ANGPTL4 E40K suppression in the derivation cohort (Fig. 6A: *R*^2^ ≈ 0.01, slope ≈ 0.33 [95% CI: −0.32–0.99], intercept ≈ −1.04 [95% CI: −1.47 to −0.62]) and validation set (Fig. 6C: *R*^2^ ≈ 0.18, slope ≈ −1.27 [95% CI: −1.94 to −0.59], intercept ≈ −1.33 [95% CI: −1.75 to −0.92]). Similarly, there was minimal concordance between EL suppression and ANGPTL4 suppression in the derivation cohort (Fig. 6B: *R*^2^ ≈ 0.03, slope ≈ −0.05 [95% CI: −0.10–0.00], intercept ≈ 0.39 [95% CI: 0.28–0.49]) and validation set (Fig. 6D: *R*^2^ ≈ 0.19, slope ≈ −0.13 [95% CI: −0.20 to −0.06], intercept ≈ 0.64 [95% CI: 0.51–0.77]). To verify that this was not an effect of E40K-specific interaction with LPL, we performed mimicry analysis using all the available metabolite-associated variants within 0.2 Mb of *ANGPTL4* (*N* = 228 SNPs) in the derivation cohort. Again, we found low concordance between genetic HL activity and the metabolite-associated loci in the *ANGPTL4* region (supplemental Fig. S7: median *R*^2^ ≈ 0.00, interquartile range ≈ 0.00–0.02, and range ≈ 0.00–0.05). We found a similar low correlation pattern for EL versus ANGPTL4 (supplemental Fig. S8: median *R*^2^ ≈ 0.08, interquartile range ≈ 0.07–0.21, and range ≈ 0.00–0.28). These results further demonstrate that the effects of ANGPTL4 on plasma lipids in humans are likely mediated exclusively by LPL.

## Discussion

In this study, we performed genetic mimicry analysis to investigate the mechanism of plasma lipid regulation by ANGPTL3, ANGPTL4, and ANGPTL8 in humans. We demonstrate that the LPL-independent effects of ANGPTL3 suppression on plasma metabolites show a striking inverse resemblance with EL suppression, suggesting that ANGPTL3 acts via not only LPL inhibition but also EL inhibition. In addition, by instrumenting ANGPTL3-ANGPTL8 complex action on systemic lipid and metabolite traits using the ANGPTL8 R59W substitution and the ANGPTL8 Q121X PTV, we find human genetic evidence suggesting an inhibitory role of the ANGPTL3-ANGPTL8 complex in both EL and LPL regulation. Interestingly, a much higher concordance was found between ANGPTL8 R59W and EL activity than between ANGPTL8 R59W and LPL activity, implying that the R59W substitution more profoundly impacts EL inhibition by the ANGPTL3-ANGPTL8 complex than LPL inhibition. In contrast, instrumentation of the ANGPTL3-ANGPTL8 complex action through a deleterious PTV was highly concordant with LPL enhancement, verifying that the ANGPTL3-ANGPTL8 complex act on plasma lipids in humans through LPL inhibition. Finally, our data also strongly indicate that ANGPTL4 acts exclusively via LPL.

Studies using genetically modified mice have firmly established that the inhibition of LPL by ANGPTL3 is dependent on ANGPTL8 (37). Specifically, it was found that adenovirus-mediated overexpression of ANGPTL3 did not raise plasma TG unless it was combined with overexpression of ANGPTL8. In addition, adenovirus-mediated ANGPTL8 overexpression raised plasma TG levels in wild-type mice but not ANGPTL3-deficient mice (37). Biochemical studies have shown that ANGPTL3 and ANGPTL8 form a tight complex that inhibits LPL much more strongly than ANGPTL3 alone (40, 41, 43). Premature truncation of ANGPTL8 prevents complex formation with ANGPTL3, indicating that the C-terminal end is required for complex formation (66). The ANGPTL3-ANGPTL8 complex is secreted by the liver and can be detected in human plasma at a level that is approximately tenfold lower than free ANGPTL3 (43). Thus far, human genetic evidence supporting a dependency between ANGPTL3 and ANGPTL8 in plasma lipid regulation has been lacking. In our genetic mimicry analysis, we found that ANGPTL3 suppression only moderately mimicked LPL enhancement. However, the protein-truncating *ANGPTL8* variant showed very high LPL mimicry, strongly suggesting that in humans, ANGPTL3 and ANGPTL8 act together in the regulation of LPL activity and plasma TG.

Current therapies to lower plasma lipids by targeting ANGPTL3 are focused on ANGPTL3 alone. Considering that the ANGPTL3-ANGPTL8 complex is the actual functional unit in plasma lipid regulation, which is confirmed here by human genetics, an alternative approach for lipid lowering is to target the ANGPTL3-ANGPTL8 complex. Recently, Balasubramaniam *et al.* (76) described a new antibody that binds the ANGPTL3-ANGPTL8 complex and blocks ANGPTL3-ANGPTL8-mediated LPL inhibition in vitro by targeting the same leucine zipper-containing epitope recognized by LPL and apolipoprotein A-V. In mice, this anti-ANGPTL3-ANGPTL8 antibody markedly lowered plasma TG levels. Based on our results, it can be argued that targeting the ANGPTL3-ANGPTL8 complex might be a more effective approach to lower plasma TG and LDL in humans than targeting ANGPTL3 alone. In this light, it is relevant to mention that the clinical development of an antisense oligonucleotide targeting ANGPTL3 (vupanorsen) was recently discontinued because of the limited magnitude of non-HDL-C and TG reduction as well as increased liver enzymes and liver fat (77).

Previous genetic mimicry analysis of ANGPTL3 suppression and LPL enhancement suggests the involvement of other pathways besides LPL inhibition in ANGPTL3 action (50). Our data strongly point to EL as an additional target of ANGPTL3, thereby providing direct human genetic evidence supporting the role of ANGPTL3 as an EL inhibitor. Data collected in LDLR-deficient mice indicate that enhanced EL activity accounts for the lowering of plasma LDL-C levels upon ANGPTL3 inactivation (44, 45), leading to the proposition that EL mediates the LDL-lowering effect of ANGPTL3-inactivating antibodies in patients with homozygous familial hypercholesterolemia (78). Currently, conflicting biochemical data exist on whether ANGPTL3 alone or the ANGPTL3-ANGPTL8 complex is a better EL inhibitor. According to Davies *et al.* (47), inhibition of EL by ANGPTL3 is not modulated by ANGPTL8. By contrast, Chen *et al.* (46) found that ANGPTL3-ANGPTL8 more potently inhibits EL than ANGPTL3 alone. Our genetic mimicry analysis suggests that in addition to regulating LPL, the ANGPTL3-ANGPTL8 complex is also involved in EL regulation. Our data thus support the notion that the role of ANGPTL3-ANGPTL8 goes beyond the regulation of LPL and plasma TG.

Intriguingly, the genetic mimicry analysis revealed a very high concordance between ANGPTL8 R59W and EL activity, whereas there was no concordance between ANGPTL8 R59W and LPL activity, suggesting the ANGPTL8 R59W substitution in the ANGPTL3-ANGPTL8 complex affects EL inhibition by ANGPTL3-ANGPTL8 much more strongly than LPL inhibition. According to the artificial intelligence-based 3-D protein structure prediction database AlphaFold (DeepMind Technologies Ltd; European Bioinformatics Institute), R59W is part of a long alpha helix in the ANGPTL8 structure (79). This helix is not expected to be disrupted by a change from arginine to tryptophan. Rather, it can be hypothesized that R59 interacts with specific residues in EL but not LPL and that this interaction is disrupted by substituting tryptophan. In contrast, Sylvers-Davies *et al.* (47) found that the R59W substitution altered the ability of ANGPTL3-ANGPTL8 to bind and inhibit LPL but not EL. Additional biochemical studies are necessary to further investigate the impact of the R59W mutation on ANGPTL8 structure and function.

A preferential impact of R59W on EL inhibition is supported by the observation that carriers of the R59W mutation have lower plasma HDL-C levels and unchanged plasma TGs (37, 80). The association between ANGPTL8 R59W and plasma lipids is somewhat complicated by the fact that R59W carrier status is associated with higher plasma ANGPTL8 levels (81). In addition, homozygous R59W carriers have higher plasma ANGPTL3 levels. Whereas *ANGPTL8* R59W was highly concordant with EL activity, the *ANGPTL8* Q121X PTV was highly LPL specific. It can be hypothesized that Q121X may interfere with the formation of the ANGPTL3-ANGPTL8 complex or lead to the loss of regions in ANGPTL8 critical for LPL inhibition. Consistent with a primary impact on LPL activity, Q121X and other *ANGPTL8* PTV carriers exhibit significantly lower plasma TG and higher HDL-C levels compared with noncarriers (26, 82).

Consistent with a previous report (50), we found that ANGPTL4 suppression and LPL enhancement have very similar metabolic effects, strongly suggesting that ANGPTL4 impacts plasma lipids exclusively via LPL. Moreover, we did not find any evidence for ANGPTL4 action on plasma lipids through HL or EL. How can these data be reconciled with the finding that ANGPTL4 may inhibit HL in mice and is a very good EL inhibitor in vitro? (12, 24, 46). Concerning HL, it is possible that ANGPTL4 only targets HL in mice and not in humans. Concerning EL, one possibility is that the E40K variant in ANGPTL4 does not influence the inhibition of EL by ANGPTL4. However, the E40K variant destabilizes the protein and leads to lower plasma ANGPTL4 levels, thus representing a general loss-of-function variant. In addition, while such a scenario could be envisaged for one particular variant, it is unlikely that it would apply to all ANGPTL4 variants studied. Another possible explanation is that the biochemical inhibition of EL by ANGPTL4 does not reflect a physiological scenario, for example, because the two proteins never physically meet each other. Future studies should try to explain the discrepancy between the genetic mimicry analysis and mouse and in vitro studies on the specificity of ANGPTL4 action toward LPL.

A major strength of this study is the huge sample size. Nevertheless, statistical power was comparatively low for the relatively uncommon variants (allele frequency <1%) in the validation set. This may affect the results in the validation analyses because the greater random error (standard errors) gave greater residuals when running regressions using the effect point estimates (see supplemental Fig. S9 for technical details). In addition, effect estimates derived from large-scale GWASs are often independent of the confounding effects that can bias estimates in observational epidemiology because of the random transmission of alleles from parents to their offspring at meiosis. Although population stratification and assortative mating are common confounders for certain phenotypes in GWASs, a recent within-sibship GWAS established that this was not the case for clinical lipid measurements in the UK Biobank (83). A limitation of this study is that we could not stratify our analyses by fasting status. Different feeding states could determine how genetically instrumented ANGPTL3, ANGPTL4, and ANGPTL3-ANGPTL8 complex action affect plasma lipids. However, the main findings from the nonfasted derivation cohort were replicated in the overnight-fasted validation set (Fig. 1, Fig. 2, Fig. 3, Fig. 5, Fig. 6). Also, a cross-cohort analysis reproduced the concordances and discordances presented in this article (supplemental Fig. S10). Another limitation was that it was not possible to perform mimicry analysis for the ANGPTL8 PTV in the validation set because of the lack of available genotype data for ANGPTL8 PTVs.

Genetic mimicry analysis predicted that drugs targeting HMG-CoA reductase and PCSK9 act on the same pathway (through LDLR) (51). However, the conditional variance explained that statistic (*R*^2^) used in the genetic mimicry analysis can only tell about the direction of effects, not the magnitude of effects. The magnitude of the effects cannot be concluded from the effects of individual instruments, since pharmacological suppression of target proteins is often at least one order of magnitude greater than genetical suppression.

In summary, our genetic mimicry analysis provides clear evidence that the biochemical pathways previously described in vitro and in rodents are operating in humans. We find that the ANGPTL3-ANGPTL8 complex regulates plasma lipid levels by impacting LPL and EL, whereas ANGPTL4 regulates plasma lipid levels exclusively via LPL. Targeting ANGPTL3-ANGPTL8 and ANGPTL4 has the potential to effectively lower plasma lipids and thereby reduce cardiovascular risk in humans.

## Data Availability

All data used in this article are publicly accessible. GWAS summary statistics for the *LPL*, *ANGPTL3*, *ANGPTL4*, *ANGPTL8* R59W, *LIPG*, and *LIPC* variant effects on metabolic traits were obtained on March 2, 2022 using the University of Bristol MRC Integrative Epidemiology Unit open GWAS infrastructure (84). GWAS summary statistics for the *ANGPTL8* Q121X coding mutation were provided to us by the authors of a recent study (55) and are available in the supplemental appendix (supplemental Table S6). For the colocalization analysis, GTEx v7 liver and whole blood tissue gene expression data were retrieved from the GTEx portal on March 1, 2022. For Table 2, on February 7, 2022, we obtained summary statistics for the genetic instruments on clinical lipids (TG, LDL-C, and HDL-C) and coronary artery disease from the combined UK Biobank and CARDIoGRAMplusC4D GWAS analyses (56, 57). Table 2 data on *ANGPTL8* Q121X coding variant were retrieved from Refs. (26, 58). The code used for performing the analyses in this article can be accessed through an online code repository upon publication (www.github.com/fredlandfors/lipase_genmimicry; Archive (DOI): https://doi.org/10.5281/zenodo.7311601).

## Supplemental Data

This article contains supplemental data (48, 49, 64, 85, 86, 87, 88).

## Conflict of interest

The authors declare that they have no conflicts of interest with the contents of this article.
