# Supplementary material for: Genetic Mimicry Analysis Reveals the Specific Lipases Targeted by the ANGPTL3-ANGPTL8 Complex and ANGPTL4
Source: J Lipid Res. 2022 Nov 11;64(1):100313. doi: 10.1016/j.jlr.2022.100313 (PMC9852701; doi:10.1016/j.jlr.2022.100313)
Supplement: Supplementary appendix [file mmc1.docx]

**Supplemental note**

[TECHNICAL NOTE 2](#_Toc121328020)

[SUPPLEMENTAL FIGURE 1 9](#_Toc121328021)

[SUPPLEMENTAL FIGURE 2 10](#_Toc121328022)

[SUPPLEMENTAL FIGURE 3 11](#_Toc121328023)

[SUPPLEMENTAL FIGURE 4 12](#_Toc121328024)

[SUPPLEMENTAL FIGURE 5 13](#_Toc121328025)

[SUPPLEMENTAL FIGURE 6 14](#_Toc121328026)

[SUPPLEMENTAL FIGURE 7 15](#_Toc121328027)

[SUPPLEMENTAL FIGURE 8 16](#_Toc121328028)

[SUPPLEMENTAL FIGURE 9 17](#_Toc121328029)

[SUPPLEMENTAL FIGURE 10 18](#_Toc121328030)

[REFERENCES 19](#_Toc121328031)

# **TECHNICAL NOTE**

**Assumptions of genetic mimicry analysis**

Genetic mimicry analysis measures the *concordance of the* *direction of effects* of genetic variation on a select number of phenotypes. If the concordance measure is very high when using several different phenotypes, it would be more likely that the target proteins act on the same pathway and that they may interact with each other. Conversely, less concordance would indicate that the target proteins do not act exclusively through the same pathway.

Within this framework, genetic variants can serve as *instruments* for the biological function of a target protein. This is of interest to evaluate the risk of off-target effects when developing gene-silencing therapies that target regulator proteins of major plasma enzymes. As a *case-in-point*, the effects of genetic suppression of *HMGCR* and *PCSK9* showed perfect concordance with the effects of statins and PCSK9 inhibitors (1, 2). Both HMGCR and PCSK9 are known to act on plasma lipids through the LDL receptor pathway. This exemplifies that the effects of natural gene suppression can be used to evaluate the effects of pharmacological gene suppression.

To fulfill the *instrumental variable criterion* of enzyme or protein enhancement or suppression, the genetic variants should have well-defined biological effects, such as 1) cis-acting loci that are associated with increased or decreased transcription of the target gene, 2) gain- or loss-of-function missense mutations that alter the function of the target protein, 3) protein-truncating variants/nonsense mutations that codes for a premature stop codon, leading to translation of a shortened protein, or suppressed transcription due to nonsense-mediated mRNA decay.

Genetic mimicry analysis shares several features with mendelian randomization analysis. However, the purpose of the use of *instrumental variables (IVs)* is different. In bivariable mendelian randomization analysis, genetic variant(s) are used to estimate the effect of a risk factor on an outcome. In bivariable genetic mimicry analysis, many phenotypes are used to estimate the concordance of effects between two different proteins, using a single genetic IV for each protein. The schematics of mendelian randomization analysis and genetic mimicry analysis are summarized in *Figures T1-T3*.

**Figure T1. Bivariable mendelian randomization analysis.** Schematic representation with methodological summary for a simple case. It should not be interpreted as a formal directed acyclic graph.

The aim of mendelian randomization analysis is to estimate the causal effect of risk factor **X** on outcome **Y** by usage of genetic variant(s) **Z** as genetic IVs (**Figure T1**). This avoids confounding by the unmeasured confounders U. For valid inference that X causes Y, the variables must follow the core assumptions of IV analysis (3, 4). To compare the different methods, they are stated here:

1. *Relevance*: The IV **Z** is associated with risk factor **X**.
2. *Exclusion* *restriction*: **Z** does not affect outcome **Y** except through **X**.
3. *Marginal exchangeability*: **Z** and **Y** do not share causes.

To identify a well-defined casual parameter where the IV estimates the average causal effect of **X** on **Y** in a whole population, or in a subpopulation of compliers, an additional fourth *homogeneity* or *monotonicity of treatment effects* condition is required to hold. Its implications for what causal conclusions can be drawn varies depends on the causality of the instrument (5).

**Bivariable genetic mimicry analysis**

**Figure T2. Bivariable genetic mimicry analysis.** This is a schematic representation. It should not be interpreted as a formal directed acyclic graph.

In bivariable genetic mimicry analysis, the objective of the analysis is to measure the degree of concordance between enhancement/suppression of proteins **X_1_, X_2_** (e.g., enzyme/regulators) on phenotypes **Y_1_, …, Y_n_**. Here, we use genetic variants **Z_1_, Z_2_** as instrumental variables for the *direction of the effects* of **X_1_, X_2_** on **Y_1_, …, Y_n_** (**Figure T2**). The effects of **X_i_** conditional on **Z_i_** does not need to be estimated directly as we are measuring the concordance between the direction of effects.

To correctly estimate the concordance of the enhancement or suppression of X_1_ and X_2_, the IVs must follow these assumptions:

1. *Relevance*: The IVs **Z_1_**, **Z_2_** must be associated with altered biologic activity of target proteins **X_1_, X_2_**, respectively. Also, this association must be concordant with the direction of effects by enhancement or suppression of proteins **X_1_, X_2_**.
2. *Exclusion restriction*: **Z_1_**, and **Z_2_** does not affect outcomes **Y_1_, …, Y_n_** except through their respective target proteins **X_1_, X_2_**.
3. *Marginal exchangeability*: **Z_1_,** and **Z_2_** does not share causes with **Y_1_, …, Y_n_**.

The first condition does not need further motivation. The second and third conditions are alike those of mendelian randomization analysis, i.e., the variant **Z_i_** can only affect outcomes **Y_1_, …, Y_n_** through its effect on the target protein **X_i_**. These could be violated by several types of confounding factors, such as **Z_1_** and **Z_2_** being in linkage disequilibrium, unbalanced pleiotropy, or population stratification bias in the original GWAS. However, the assumptions (ii-iii) should not be misread as if the genetic mimicry IVs would need to be exclusive for the different biological pathways of interest. Instead, the method can be used to estimate the degree of pathway exclusivity under certain conditions, which are described below.

If assumptions (*i-iii)* hold, then the method correctly estimates the concordance between the effects of **X_1_** and **X_2_**. However, to conclude that proteins **X_1_** and **X_2_** act through the same pathway *if they are highly concordant* (e.g., R^2^ > 0.90), additional assumptions not directly related to the IVs would be required. (*iv)* A fourth assumption would be that the effects of other unmeasured pathways **U** on **Y_1_, …, Y_n_** could not be parallel to the effects of **X_1_** on **Y_1_, …, Y_n_**. We could call this a *convergence* assumption ($\boldsymbol{\beta∦\delta}$).
Additionally, if **X_1_** and **X_2_** are not highly concordant (e.g., R^2^ = 50 %), and if the effects of **U** and **X_1_** were *orthogonal* (alternative assumption *iv)*, then the regression would estimate how much of **X_2_** is mediated through **X_1_**, as $\boldsymbol{\beta\perp\delta}$ infers that $\boldsymbol{\beta\perp}\boldsymbol{\alpha}^{\boldsymbol{**}}$. To evaluate the validity of these assumptions, one could envision that an investigator would investigate the relationship between **X_1_** and a set of candidate **U** variables based on previous knowledge about the pathway of interest. For example, if **X_1_** is LPL, the concordance between the effects of LPL and other major plasma lipases **U**_1_, **U_2_** (EL, HL) would be investigated. If they are not correlated (they are not, see **Figures T6-T8**), then the assumption would hold for this set of proteins.

Another core assumption not related to the IVs would be that (*v*) the set of phenotypes **Y_1_, …, Y_n_** must be sufficiently *representative* of the parameters that operate in the biological system of interest. This fifth assumption would have to be evaluated entirely based on previous biological knowledge.

**Multivariable genetic mimicry analysis**

**Figure T3. Multivariable genetic mimicry analysis.** This is a schematic representation. It should not be interpreted as a formal directed acyclic graph.

The analysis could the extended into the multivariable case, using a single dependent variable and multiple independent variables (**Figure T3**). If proteins **X_1_, …, X_n_** meet the assumptions (*i-iii)*, then the R^2^ will estimate the concordance between **X_1_** and **X_2_** conditional on **X_3_, …, X_n_**. If the concordance between **X_1_** and **X_2_** is intermediate (e.g., R^2^= 0.50), and assumption (*iv)* hold for both **X_1_**, and **X_3_** given **X_1_** (i.e., $\boldsymbol{\beta\perp\delta}\boldsymbol{, and}\boldsymbol{\beta\perp}\boldsymbol{\gamma}^{\boldsymbol{**}}$). Then, the multivariable approach can be useful to identify additional **U** through which **X_2_** may act. If the conditional explained variance increases (measured by R^2^) by adding **X_3_** to the model, this would indicate that **X_3_** accounts for the residual variance from the bivariable model (**X_1_ ~ [**conditional on] **X_2_**). An example of this situation is given in the next section.

**Schematic representations of bivariable and multivariable models from the manuscript**

**Figure T4. Example of bivariable genetic mimicry analysis.** LPL vs. ANGPTL4 in the derivation set, where LPL activity was instrumented through the rs115849089-A eQTL variant. ANGPTL4 activity was instrumented through the rs116843064-A E40K coding variant.

The estimated mimicry of ANGPTL4 with LPL on NMR metabolites **Y_1,…_,Y_248_** was $R^{2}=0.99$. Then ANGPTL4 acts through LPL, if the linear equations of $\boldsymbol{E}\left[ \boldsymbol{Metabolites} \right| \boldsymbol{LPL}]\boldsymbol{∦}E\left[ \mathbf{M}\boldsymbol{etabolites} \right|\boldsymbol{U]}$, or $\boldsymbol{\beta∦\delta}$. Additionally, if the linear equations of $\boldsymbol{E}\left[ \boldsymbol{Metabolites} \right| \boldsymbol{LPL}]\boldsymbol{\perp}E\left[ \mathbf{M}\boldsymbol{etabolites} \right|\boldsymbol{EL]}$ and $\boldsymbol{E}\left[ \boldsymbol{Metabolites} \right| \boldsymbol{LPL}]\boldsymbol{\perp}E\left[ \mathbf{M}\boldsymbol{etabolites} \right|\boldsymbol{HL]}$**,** or $i.e.\boldsymbol{,}\boldsymbol{\beta\perp\delta}$, then ANGPTL4 exclusively acts through LPL.

**Figure T5. Example of multivariable genetic mimicry analysis.** LPL vs. ANGPTL4 in the derivation set, where LPL activity was instrumented through the rs115849089-A eQTL variant. ANGPTL3 activity was instrumented through the rs11207977-T eQTL. ANGPTL8 R59W activity was instrumented through the rs2278426-T R59W coding variant.

The estimated mimicry of ANGPTL3 with LPL on NMR metabolites **Y_1,…_,Y_248_** was $R^{2}=0.58$. By adding ANGPTL8 R59W to the model, the residual variation in the LPL ~ ANGPTL3 relationship is accounted for, if the linear equation estimating the conditional $\boldsymbol{E}\left[ \boldsymbol{Metabolites} \right| \boldsymbol{LPL, ANGPTL}\boldsymbol{3}]\boldsymbol{\perp}E\left[ \mathbf{M}\boldsymbol{etabolites} \right|\boldsymbol{HL]}$**,** or $\boldsymbol{\beta\perp}\boldsymbol{\gamma}^{\boldsymbol{**}}$. This was because ANGPTL8 R59W was highly correlated to EL activity, which is the additional pathway through which ANGPTL3 act on plasma lipids.

**Fig T6. Validity of the assumption that $\left[ \boldsymbol{Metabolites} \right| \boldsymbol{LPL}]\boldsymbol{\perp}\left[ \mathbf{M}\boldsymbol{etabolites} \right|\boldsymbol{EL]}$.**

**Fig T7. Validity of the assumption that** $\left[ \boldsymbol{Metabolites} \right| \boldsymbol{LPL}]\boldsymbol{\perp}\left[ \mathbf{M}\boldsymbol{etabolites} \right|\boldsymbol{HL]}$

**Fig T8. Validity of the assumption that** $\left[ \boldsymbol{Metabolites} \right| \boldsymbol{LPL, ANGPTL}\boldsymbol{3}]\boldsymbol{\perp}\left[ \mathbf{M}\boldsymbol{etabolites} \right|\boldsymbol{HL]}$

# **SUPPLEMENTAL FIGURE 1**

**Figure S1. Colocalization analysis of the *LPL rs115849089* region using genetic variants within a ± 200,000 base pair window.** The colocalization test gave a 70.0 % posterior probability of plasma total triglycerides and LPL expression in whole blood sharing a single casual variant in the region.

# **SUPPLEMENTAL FIGURE 2**

**Figure S2. Sensitivity analysis of *LPL rs115849089-A* versus *LPL rs1801177-A*.** LPL enhancement through increased LPL transcription showed a high degree of concordance with genetic inhibition of LPL via the D36N missense mutation (R^2^ ≈ 0.96, slope ≈ -0.94 [95% CI: -0.97 – -0.90], intercept ≈ -0.05 [95% CI: -0.08 – -0.03]). The variants were not in significant linkage disequilibrium (R^2^  = 0.033) in a British population, as determined by the LDpair tool supplied by the NIH NCI Division of Cancer Epidemiology and Genetics (6).

# **SUPPLEMENTAL FIGURE 3**

**Figure S3. Sensitivity analysis of our main results using *LPL rs1801177-A* D36N in the UK biobank derivation cohort (N = 110,058 – 115,078).** Genetic instrumentation of LPL inhibition using the D36N functional variant gave essentially the same (but inverse) results as when instrumenting LPL enhancing through the eQTL variant *LPL rs115849089-A* (See ***Figures 1-2, 4-5*** in the manuscript).

# **SUPPLEMENTAL FIGURE 4**

**Figure S4. Colocalization analysis of the *ANGPTL3 rs11207977* region using genetic variants within a ± 200,000 base pair window.** The colocalization test gave a 94.1 % posterior probability of serum total triglycerides and ANGPTL3 expression in liver sharing a single casual variant in the region.

# **SUPPLEMENTAL FIGURE 5**

**Figure S5. Linkage disequilibrium of ANGPTL8 rs2278426 using a 1 mega base pair window.** Rs2278426 was not in significant LD with any exonic LDLR variants.

# **SUPPLEMENTAL FIGURE 6**

**Figure S6. LPL activity mimicry analysis of all available metabolite-associated variants (N = 228 SNPs) in the *ANGPTL4* region (chr 19:8,229,011 – 8,629,011).**  The analysis was conducted in the derivation cohort (N subjects = 110,058 – 115,078, N metabolites = 248). The concordance with LPL enhancement was high (median R^2^ ≈ 0.93, interquartile range ≈ 0.86 – 0.94, range ≈ 0.79 – 0.99). LD indicates variant linkage disequilibrium with *ANGPTL4 rs116843064-A* [E40K] measured by r^2^. LD was derived from the 1000 Genomes matrix using a European reference population. Grey-coloured dots indicates that information on LD was missing from the LD matrix. To provide chromosomal position context, *Figures S6-S8* was annotated with UCSC hg19 gene track data. Reuse of the gene track data for these *ANGPTL4* region analyses was intentional.

# **SUPPLEMENTAL FIGURE 7**

**Figure S7. Hepatic lipase (HL, *LIPC*) activity mimicry analysis of all available metabolite-associated variants (N = 228 SNPs) in the *ANGPTL4* region (chr 19:8,229,011 – 8,629,011).**  The analysis was conducted in the derivation cohort (N subjects = 110,058 – 115,078, N metabolites = 248). The concordance with HL inhibition was low (median R^2^ ≈ 0.00, interquartile range ≈ 0.00 – 0.02, range ≈ 0.00 – 0.05). LD indicates variant linkage disequilibrium with *ANGPTL4 rs116843064-A* [E40K] measured by r^2^. LD was derived from the 1000 Genomes matrix using a European reference population. Grey-coloured dots indicates that information on LD was missing from the LD matrix. To provide chromosomal position context, *Figures S6-S8* was annotated with UCSC hg19 gene track data. Reuse of the gene track data for these *ANGPTL4* region analyses was intentional.

# **SUPPLEMENTAL FIGURE 8**

**Figure S8. Endothelial lipase (EL, *LIPG*) activity mimicry analysis of all available metabolite-associated variants (N = 228 SNPs) in the *ANGPTL4* region (chr 19:8,229,011 – 8,629,011).**  The analysis was conducted in the derivation cohort (N subjects = 110,058 – 115,078, N metabolites = 248). The concordance with EL inhibition was low (median R^2^ ≈ 0.08, interquartile range ≈ 0.07 – 0.21, range ≈ 0.00 – 0.28). LD indicates variant linkage disequilibrium with *ANGPTL4 rs116843064-A* [E40K] measured by r^2^. LD was derived from the 1000 Genomes matrix using a European reference population. Grey-coloured dots indicates that information on LD was missing from the LD matrix. To provide chromosomal position context, *Figures S6-S8* was annotated with UCSC hg19 gene track data. Reuse of the gene track data for these *ANGPTL4* region analyses was intentional.

# **SUPPLEMENTAL FIGURE** **9**

**Figure S9. Genetic mimicry analysis requires precise effect estimates.** We used the covariance matrix from one of the author’s previous papers where the NMR metabolic parameters were directly measured (7). We inserted genotype-metabolite effects and added random noise that would match the genotype-metabolite effect sizes (exact match) and standard errors (approximate match, it’s not possible to copy the real *error* term) of the LPL eQTL and ANGPTL4 E40K (genetic mimicry R^2^ = 0.99) variants from the UK biobank derivation cohort. Using this setup, we simulated up to 120000 participants. Then, we bootstrapped the ‘genetic mimicry’ R^2^ between the variants using 1000 random resamples. This procedure was repeated for each sample size level (N = 100 – 120,000). The distributions of the R^2^ values are displayed as two-sided ‘violin’ densities. Be reminded that the “true” R^2^ always was 99 %.

# **SUPPLEMENTAL FIGURE 10**

**Figure S10. Cohort cross-over genetic mimicry analysis reproduces the concordances and discordances that were performed for the main manuscript. ‘**Deriv.’ and ‘Valid.’ denotes the derivation cohort and validation set, respectively. ‘Deriv. (y) vs. valid. (x)’ denotes that the derivation set effect estimates were used as the *dependent* (y-axis) variable, and that the validation set effect estimates were used as the *independent* (x-axis) variable. ‘Valid. (y) vs. deriv. (x)’ denotes the opposite relationship.

# **REFERENCES**

1. Würtz P, Wang Q, Soininen P, Kangas AJ, Fatemifar G, Tynkkynen T, et al. Metabolomic Profiling of Statin Use and Genetic Inhibition of HMG-CoA Reductase. J Am Coll Cardiol. 2016;67(10):1200-10.

2. Sliz E, Kettunen J, Holmes MV, Williams CO, Boachie C, Wang Q, et al. Metabolomic consequences of genetic inhibition of PCSK9 compared with statin treatment. Circulation. 2018;138(22):2499-512.

3. Greenland S. An introduction to instrumental variables for epidemiologists. Int J Epidemiol. 2000;29(4):722-9.

4. Didelez V, Sheehan N. Mendelian randomization as an instrumental variable approach to causal inference. Stat Methods Med Res. 2007;16(4):309-30.

5. Swanson SA, Hernán MA. The challenging interpretation of instrumental variable estimates under monotonicity. Int J Epidemiol. 2018;47(4):1289-97.

6. Machiela MJ, Chanock SJ. LDlink: a web-based application for exploring population-specific haplotype structure and linking correlated alleles of possible functional variants. Bioinformatics. 2015;31(21):3555-7.

7. Kovrov O, Landfors F, Saar-Kovrov V, Näslund U, Olivecrona G. Lipoprotein size is a main determinant for the rate of hydrolysis by exogenous LPL in human plasma. J Lipid Res. 2022;63(1):100144.
